# Serotonin promotes the proliferation of serum-deprived hepatocellular carcinoma cells via upregulation of FOXO3a

**DOI:** 10.1186/1476-4598-12-14

**Published:** 2013-02-19

**Authors:** Chao Liang, Wei Chen, Xiao Zhi, Tao Ma, Xuefeng Xia, Hao Liu, Qi Zhang, Qida Hu, Yun Zhang, Xueli Bai, Tingbo Liang

**Affiliations:** 1 Department of Surgery, the Second Affiliated Hospital, Zhejiang University School of Medicine, 88 Jiefang Road, Hangzhou 310009, China

**Keywords:** Hepatocellular carcinoma, Serotonin, FOXO3a, 5-HT2B receptor, Cell proliferation

## Abstract

**Background:**

Peripheral serotonin is involved in tumorigenesis and induces a pro-proliferative effect in hepatocellular carcinoma (HCC) cells; however, the intracellular mechanisms by which serotonin exerts a mitogenic effect remain unclear. In this research, we examined whether FOXO3a, a transcription factor at the interface of crucial cellular processes, plays a role downstream of serotonin in HCC cells.

**Results:**

The cell viability and expression of FOXO3a was assessed in three HCC cell lines (Huh7, HepG2 and Hep3B) during serum deprivation in the presence or absence of serotonin. Serum free media significantly inhibited HCC proliferation and led to reduced expression and nuclear accumulation of FOXO3a. Knockdown of FOXO3a enhanced the ability of serum deprivation to inhibit HCC cells proliferation. And overexpression of non-phosphorylated FOXO3a in HCC cells reversed serum-deprivation-induced growth inhibition. Serotonin reversed the serum-deprivation-induced inhibition of cell proliferation and upregulated FOXO3a in Huh7 cells; however, serotonin had no effect on the proliferation of serum-deprived HepG2 or Hep3B cells. In addition to proliferation, serotonin also induced phosphorylation of AKT and FOXO3a in serum-deprived Huh7 cells but not in HepG2 and Hep3B cells. However, the phosphorylation of FOXO3a induced by serotonin did not export FOXO3a from nucleus to cytoplasm in serum-deprived Huh7 cells. Consequently, we demonstrated that serotonin promoted the proliferation of Huh7 cells by increasing the expression of FOXO3a. We also provide preliminary evidence that different expression levels of the 5-HT2B receptor (5-HT_2B_R) may contribute to the distinct effects of serotonin in different serum-deprived HCC cells.

**Conclusions:**

This study demonstrates that FOXO3a functions as a growth factor in serum-deprived HCC cells and serotonin promotes the proliferation of serum-deprived HCC cells via upregulation of FOXO3a, in the presence of sufficient levels of the serotonin receptor 5-HT_2B_R. Drugs targeting the serotonin-5-HT_2B_R-FOXO3a pathway may provide a novel target for anticancer therapy.

## Background

Liver cancer remains the fifth most common malignancy in men and the eighth most common malignancy in women worldwide. The number of new cases of liver cancer is still increasing every year, and the disease has a poor prognosis and high rate of recurrence [1]. Many cytokines contribute to the processes of initiation and development in hepatocellular carcinoma, such as interleukin-2 (IL-2), transforming growth factor ß (TGF-ß), vascular endothelial growth factor (VEGF) and insulin growth factor-1 (IGF-1).

Serotonin, also known as 5-hydroxytryptamine (5-HT), is a neurotransmitter and vasoactive substance [2,3]. Apart from its important roles in the central nervous system (CNS), many studies have revealed that serotonin exerts various extra-neuronal physiological and pathophysiological functions. Peripheral serotonin is mostly stored in platelets [2]. Serotonin can manifest pro-proliferative or anti-apoptoticeffects in different tissues via binding to its receptors [2,4-6]. Amongst the numerous 5-HT receptor families, the 5-HT2B receptor (5-HT_2B_R) is necessary for cell viability and proliferation [7]. 5-HT_2B_R is expressed at low levels in discrete regions of the CNS; however, it is expressed at high levels in the hepato-gastrointestinal (GI) tract, including the liver [8]. This expression pattern differs from the other type 5-HT2 receptors, which are expressed at relatively high levels in the CNS [9]. The physiological function of 5-HT_2B_R is still unclear, and there are few studies of its downstream targets [4,10]; however, 5-HT_2B_R can initiate liver regeneration and mediate hepatocyte fibrosis [11], which contributes to the genesis of hepatocellular carcinoma.

FOXO3a is a member of class O of the fork head box family of transcription factors which includes FKHR/FOXO1, FKHRL1/FOXO3, AFX/FOXO4 and FOXO6 [12,13]. FOXO3a is homologous to the *Caenorhabditis elegans* transcription factor Dauer Formation Influencing Genes-16(DAF-16), which regulates lifespan by acting as downstream of the *C. elegans* insulin receptor DAF-2 [13-16]. FOXO3a plays important roles in metabolism, cellular proliferation, stress tolerance and also possibly regulates the lifespan of mammalian cells [17-20]. Previous studies have shown that FOXO3a acts as a tumor suppressor by inducing apoptosis [18,21]; and recent researches have provided evidence that FOXO3a has multiple functions, especially with regards to aging and stress resistance [19,20,22,23].These studies have suggested that FOXO3a may exert opposing effects and orchestrate different responses, based on the extracellular stimuli or the specific cell type [12,24-26]. As FOXO3a may mediate different cellular functions, we were interested to investigate the role of FOXO3a in hepatocellular carcinoma, due to the scarce research in this field.

As a common type of cellular stress, serum deprivation can induce G0 phase cell cycle arrest and reduce the proliferation of most cell types [27-29]. However, serotonin can reverse the inhibition of proliferation in serum-deprived HCC cells, which exhibit significant growth inhibition in the absence of serotonin and eventually undergo complete necrotic death [30]. Recently, accumulating evidence has revealed that FOXO3a can ensure metabolic stability under stress conditions in various cell types [17]. Loss of FOXO3a can enhance the sensitivity of cells to stress, such as serum deprivation [23,24]; however, there are few reports on the function of FOXO3a in serum-deprived HCC cells. Therefore it was of interest to investigate the expression pattern of FOXO3a in response to serum deprivation in HCC cells. We clarified the role of FOXO3a in serum-deprived HCC cells treated with serotonin, and investigated whether FOXO3a functions as a downstream target of serotonin to modulate the proliferation of serum-deprived HCC cells.

## Materials and methods

### Cell lines and cell culture

The human hepatocarcinoma cell lines (Huh7, Hep3B and HepG2) were purchased from the Shanghai Institute for Biological Science (Shanghai, China). HepG2 and Huh7 cells were cultured in Dulbecco’s Minimal Essential Medium (DMEM; Gibco; Carlsbad, CA, USA) containing 10% fetal bovine serum (FBS; Gibco), 100 U/mL penicillin, and 100 mg/mL streptomycin. Hep3B cells were cultured in Minimal Essential Medium (MEM; Gibco) supplemented with 10% FBS and 1% penicillin/streptomycin. The cells were maintained at 37°C in 5% CO_2_ and 95% air.

### Experimental conditions

HCC cells were harvested, re-plated and incubated overnight to allow the cells to adhere. The cell cycle was synchronized by incubating the cells in serum-free medium for 24 h, then the media was replaced with media containing drugs of different concentrations as indicated. Most experiments contained three experimental groups: FBS group (media containing 10% FBS), SFM group (serum free media containing 10% phosphate-buffered saline) and the serum-free media plus serotonin (SFM+5-HT) group. Cells were pretreated in the media containing the 5-HT2B receptor antagonist SB204741 (SB204) for 30 minutes before addition of 5-HT. The results from all assays were confirmed in at least three independent experiments.

### Drugs and antibodies

Serotonin-creatinine complex (5-HT) and the serotonin 2B receptor antagonist SB204741 were purchased from Sigma-Aldrich (St. Louis, MO, USA). The total-FOXO3a (t-FOXO3a), phospho-FOXO3a (Thr32; *p*-FOXO3a), total-AKT (t-AKT), phospho-Akt (Ser473; *p*-AKT) primary antibodies for Western blotting or immunofluorescence staining were obtained from Cell Signaling (Danvers, MA, USA), HRP-conjugated secondary antibody, mouse anti-GFP antibody and mouse anti-GAPDH antibody were purchased from Kangchen Biotechnology (Shanghai, China).

### Assessment of cell viability and proliferation

Cell viability was measured using the cell count kit-8 (CCK-8; Dojindo; Kumamoto, Japan) following the manufacturer’s protocol. HCC cells were plated into 96-well plates at a density of 3000 cells/well in 100 μL media. After starvation in serum-free medium for 24 h, the media was removed and replaced with media containing different concentrations of 5-HT. The cells were cultured for the indicated times. The relative viability of HCC cells was expressed as the percentage of CCK-8 value of synchronized cells without treatments. Cell proliferation assay was performed using Click-iT 5-ethynyl-20-deoxyuridine (EdU) Imaging Kit (Invitrogen; Carlsbad, CA, USA) following the manufacturer’s protocol. After treatments described in cell viability assay, cells were incubated with 10 μM EdU for 2h at 37°C. Then, cells were fixed with 4% formaldehyde for 15 min at room temperature and permeabilized with 0.5% Triton X-100 for 20 min at room temperature. After three washes with phosphate-buffered saline (PBS), cells were incubated with 1X Click-iT reaction cocktails in dark for 30 min. HCC cells nuclei stained by Hoechst 33342 were used to cell count and visualized using an inverted fluorescence \microscope (Olympus, Tokyo, Japan). For quantification of HCC cells proliferative rate, five randomly selected views from each sample image were used to calculate the relative EdU-positive ratio.

### Transfection of siRNAs

Cells were transfected with scrambled negative control siRNA (NC-siRNA) or *FOXO3a*-siRNA (Santa Cruz Biotechnology; Santa Cruz, CA, USA) using Lipofectamine 2000 (Invitrogen) for 6 h; all experiments were performed in 24 h after transfection. The efficiency of the siRNA was tested by measuring the relative FOXO3a protein expression levels by Western blotting. Transfected cells were re-plated in 96-well plates (3000 cells/well), allowed to adhere overnight and treated as previously described before the subsequent experiments.

### Transfection of FOXO3a overexpression plasmid

The FOXO3a overexpression plasmid, pEGFP-c2-*FOXO3a*-3A, in which the three AKT phosphorylation sites are mutated, encoding constitutively active form of FOXO3a was purchased from Addgene (Cambridge, MA, USA). Cells were transfected with Negative control vector or pEGFP-c2-*FOXO3a*-3A using Lipofectamine 2000 for 12h; fluorescent images were visualized using an inverted fluorescence microscope at 24h after transfection. The transfection efficiency was tested by measuring fusion protein (EGFP-FOXO3a) expression by Western blot.

### Immunofluorescence

HCC cells were seeded into 48-well plates at a density of 5000 cells/well and treated in conditioned media for the indicated times. After treatments, cells were fixed with 4% formaldehyde before being permeabilized in ice-cold PBS containing 0.1% Triton X-100 for 10 min, blocked with 5% bovine serum albumin for 30 min at room temperature, and incubated with anti-FOXO3a primary antibody at 4°C overnight. After three washes with PBS, the cells were incubated with secondary antibody conjugated to Cy5 (Abcam; Cambridge, USA) at 4°C for 2 h followed by incubation with 4',6-diamidino-2-phenylindole (DAPI; Sigma) for 2 min at room temperature for nuclear counterstain. The immunofluorescent images were observed and captured using an inverted fluorescence microscope. The quantification of subcellular localization of FOXO3a was performed by calculating the percentage of FOXO3a nuclear localization of 100 cells from five randomly selected views of each sample image.

### Flow cytometric analysis

Pretreated Huh7, HepG2 and Hep3B cells were incubated in conditioned medium for 48 h, rinsed twice with ice-cold PBS, fixed with 70% pre-chilled ethanol at 4°C overnight, centrifuged and washed twice with PBS. Cell cycle analysis was performed by staining the DNA with propidium iodide (PI; Dawen; Shanghai, China) and flow cytometry. Quantitative cell cycle analysis was performed using the ModFit software (Verity software house; Topsham, ME, USA). Cell proliferation was expressed as the proliferation index (PI), which is the ratio of S+G2/M phase cells [31].

### Quantitative real time PCR (qRT-PCR)

Total RNA was isolated and reverse transcribed from Huh7, HepG2 and Hep3B cells in the FBS group, SFM group or 5-HT group for 48H using TriZol Reagent (Invitrogen) and Prime Script reagent RT Kit (Takara Biotechnology Co.; Dalian, China) following the manufacturer’s protocol. The primers for *FOXO3a* and *5-HT*_*2B*_*R* were designed and purchased from Takara: *FOXO3a* (forward, 5-TGCGTGCCCTACTTCAAGGATAA-3; reverse, 5-ACAGGTTGTGCCGGATGGA-3), *5-HT*_*2B*_*R* (forward, 5- CATGCATCTCTGTGCCATTTCA-3; reverse, 5-TGTTTGGGTTGTCCACATCAGTC-3). qRT-PCR was performed on the ABI 7900 Prism HT (Applied Biosystems Inc.; Shanghai, China) followed by melting curve analysis. *FOXO3a* and *5-HT*_*2B*_*R* mRNA expression was normalized to *β-actin* (forward, 5- TGGCACCCAGCACAATGAA-3; reverse, 5- CTAAGTCATAGTCCGCCTAGAAGCA-3); each treatment was assayed in triplicate.

### Western blotting

HCC cell lysates were washed twice in ice-cold PBS and resuspended in cell lysis buffer (Cell Signaling) containing protease inhibitors (Sigma). The protein concentration was quantified using the BCA Protein assay kit (Thermo Fisher Scientific Inc.; Rockford, IL, USA). The protein lysates added with loading buffer were denaturated by boiling, separated using 10% SDS-PAGE gels and then transferred to polyvinyllidenediflouride (PVDF) membranes (Millipore; Billerica, MA, USA), blocked with Tris-buffered saline (TBS) and 0.1% Tween 20 (TBS/T) containing 5% bovine serum albumin and then incubated with primary anti-t-FOXO3a, anti-*p*-FOXO3a (Thr32), anti-t-AKT, anti-*p*-AKT (Ser473), anti-GFP, anti-GAPDH antibodies at 4°C overnight. The membranes were washed three times with TBS/T, incubated with the appropriate HRP-conjugated secondary antibody for 1 h at room temperature, washed. The protein bands were developed by enhanced chemiluminescence (GE Healthcare; Piscataway, NJ, USA) and visualized using an autoradiography kit (Kodak; Rochester, NY, USA). Band densities were estimated using Image Pro Plus (Media Cybernetics, Inc.; Bethesda, MD, USA) and the relative protein expression levels were normalized to GAPDH.

### Statistical analysis

All the experiments data were shown as the mean ± SD values. Two-way analysis of variance (ANOVA) using Bonferroni’s post-hoc tests or the Student’s *t*-test were used to assess the significance of the different treatments; statistical significance was defined as *P*<0.05.

## Results

### Serotonin exhibits a pro-proliferative effect in serum-deprived Huh7 cells, but not in serum-deprived HepG2 or Hep3B cells

**Figure 1:**
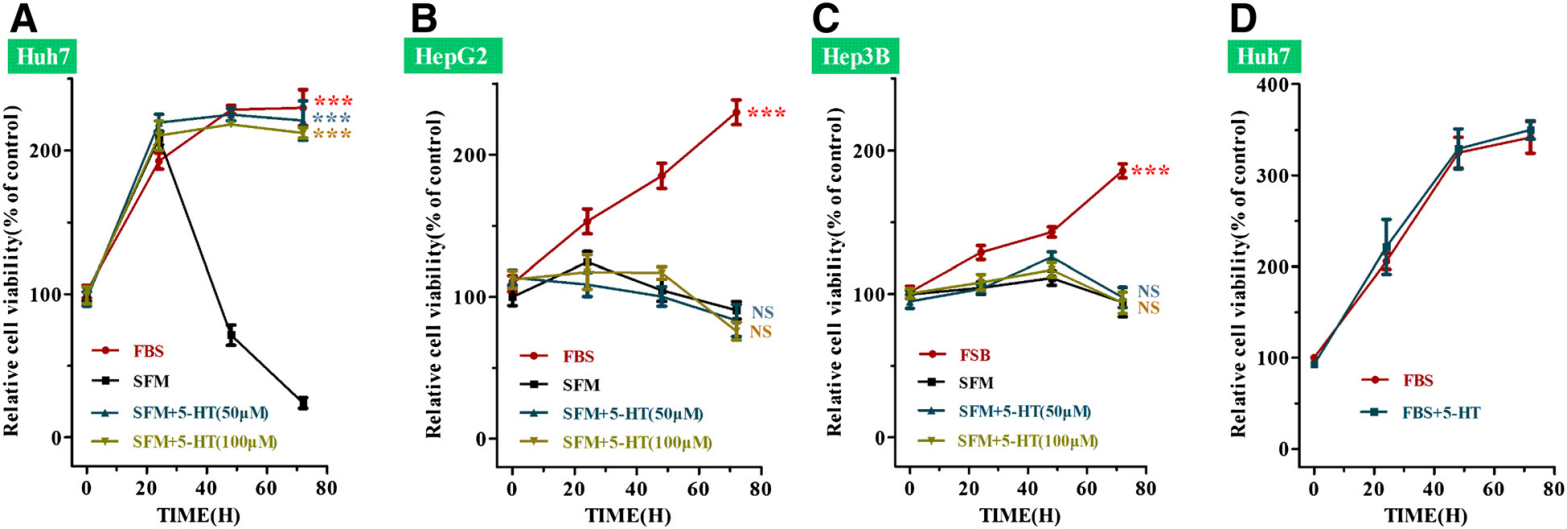
**Serotonin promotes cell proliferation in serum-deprived Huh7 cells.** (**A-C**) Mean ± SD relative viability of HCC cells cultured in media containing 10% fetal bovine serum (FBS), or serum free media (SFM) with or without serotonin (SFM + 5-HT); (**A**)Huh7 cells, (**B**)HepG2 cells, (**C**)Hep3B cells (compared with SFM, ***P < 0.001, NS denotes no significant difference, two-way ANOVA). (**D**) Mean ± SD relative viability of Huh7 cells cultured in media containing 10% FBS with or without 5-HT (50 μM); *P*>0.05, two way ANOVA.

To test if serotonin could promote the proliferation of HCC cells, we measured the cell viability of Huh7, HepG2 and Hep3B cells in the absence or presence of 10% fetal bovine serum in combination with serotonin. After 48 h serum starvation, all cell lines exhibited much lower viabilities compared to cells cultured in media containing 10% FBS (Figure 1A, B, C; SFM vs*.* FBS, ***P<0.001, two-way ANOVA). However, in the presence of serotonin, serum-deprived Huh7 cells exhibited a similar growth trend to the FBS group, and after 48 h serum deprivation, the cell viability of the serotonin serum-deprived group was almost five-fold higher than cells cultured in SFM alone (Figure 1A; SFM vs. SFM + 5-HT _(50μM)_,*** *P <* 0.001, two-way ANOVA). However, serotonin did not remarkably affect the proliferation of HepG2 or Hep3B cells in SFM (Figure 1B, C; SFM vs. SFM + 5-HT _(50μM)_, NS denotes no significant difference, two way ANOVA).

To fully understand the pro-proliferative effect of serotonin in Huh7 cells, we also investigated the effect of serotonin in media containing 10% FBS. As shown in Figure 1D, serotonin did not promote the proliferation of Huh7 cells in the presence of 10% FBS (Figure 1D; *P*>0.05, two way ANOVA).

### FOXO3a is downregulated in serum-deprived cells

**Figure 2:**
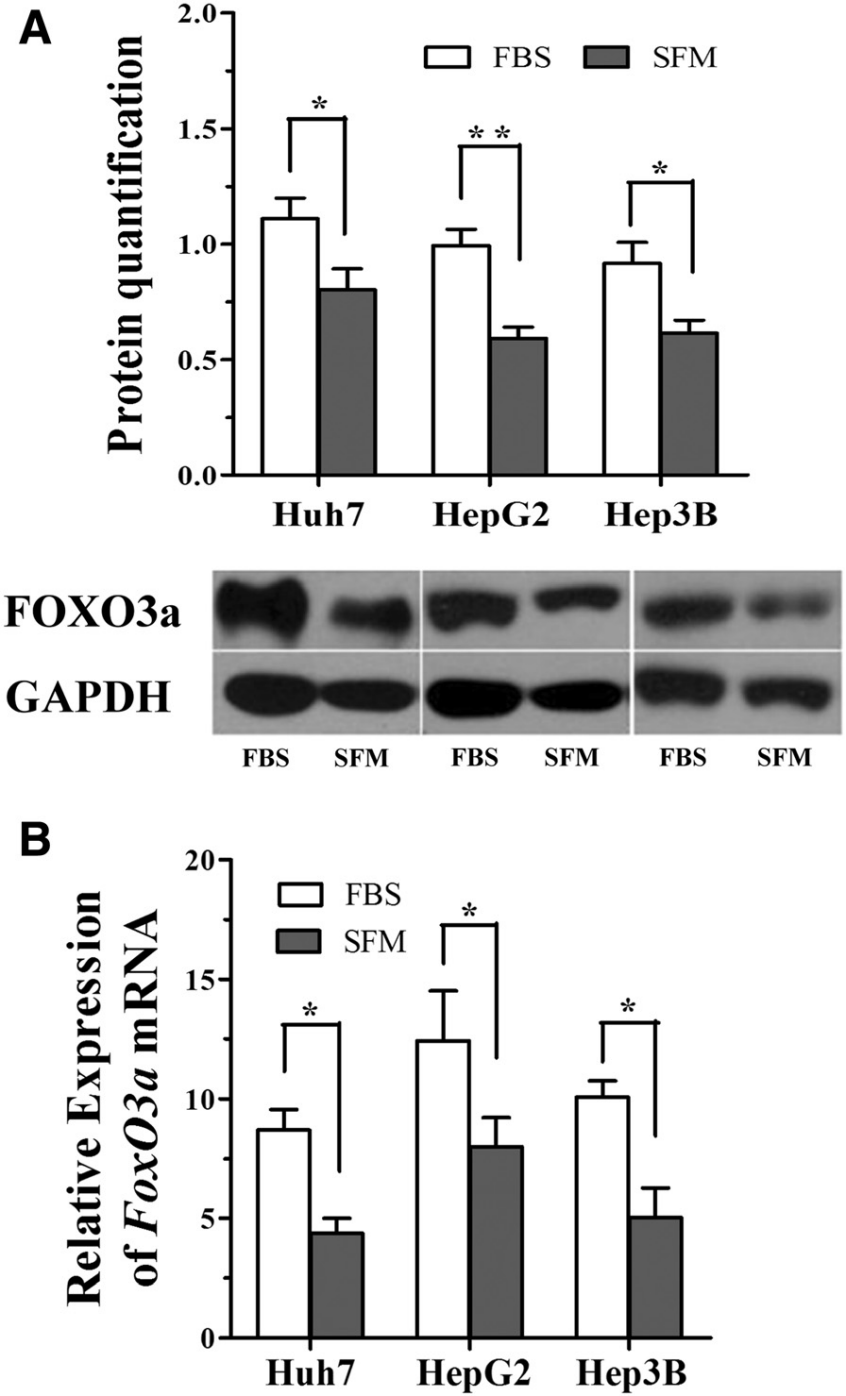
**Serum-deprivation reduces expression of FOXO3a in HCC cells.** (**A, B**) Western blot (**A**) and qRT-PCR analysis (**B**) of FOXO3a expression in HCC cells cultured in media containing 10% fetal bovine serum (FBS) or serum free media (SFM) for 48h; *P<0.05, **P<0.01, *t*-test.

As the cell proliferation rates were notably reduced in serum free conditions, we determined the effect of serum deprivation on the expression of FOXO3a. We quantified FOXO3a expression at the mRNA and protein levels in Huh7, HepG2 and Hep3B cells cultured in serum free medium for 48 h. Compared to the 10% FBS groups, the expression of FOXO3a protein significantly decreased in the serum-free groups (Figure 2A; FBS vs. SFM, *P < 0.05 for Huh7 and Hep3B, **P < 0.01 for HepG2, *t*-test;).

The real time qRT-PCR analysis displayed a similar trend to the Western blotting results (Figure 2B; FBS vs. SFM,*P < 0.05, *t*-test). These results indicated that FOXO3a may be responsible, at least in part, for the reduced cell proliferation rate induced by serum deprivation.

### FOXO3a functions as a growth factor in serum-deprived HCC cells

**Figure 3:**
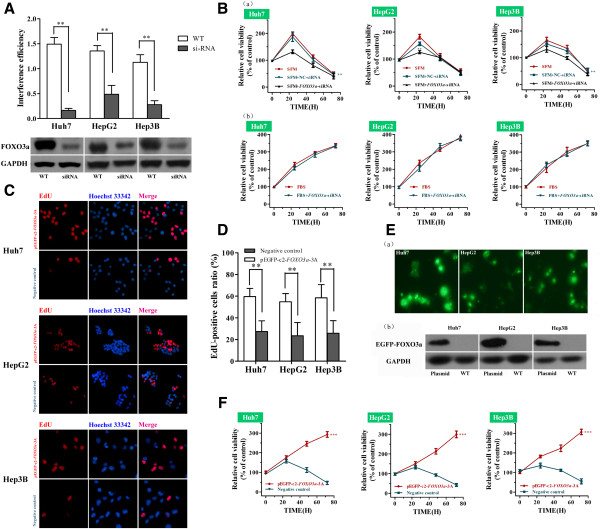
**Effect of FOXO3a on proliferation of serum-deprived HCC cells.** (**A**) Western blot analysis of the efficiency of *FOXO3a*-siRNA (siRNA) knockdown compared to control cells (WT); **P<0.01, *t*-test. (**B**) Mean ± SD relative viability of HCC cells cultured in serum free media (SFM) and transfected with negative control siRNA (NC-siRNA) or *FOXO3a*-siRNA (**a**); Huh7 and Hep3B cells, SFM+NC-siRNA vs. SFM+*FOXO3a*-siRNA, **P < 0.01, two-way ANOVA; HepG2 cells, SFM+NC-siRNA vs. SFM+*FOXO3a*-siRNA, **P < 0.01, *t-*test. (**B**) Mean ± SD relative viability of HCC cells cultured in media containing 10% fetal bovine serum (FBS) and transfected with *FOXO3a*-siRNA (**b**); all *P*>0.05, two way ANOVA. EdU staining patterns (**C**) and the relative EdU-positvie cells ratio (**D**) of HCC cells transfected with pEGFP-c2-*FOXO3a*-3A plasmid or Negative control after 48h serum deprivation; **P<0.01, *t*-test. (**E**) Fluorescent images of HCC cells transfected with pEGFP-c2-*FOXO3a*-3A plasmid (**a**) and Western blot analysis of the efficiency of pEGFP-c2-*FOXO3a*-3A plasmid (plasmid) transfection compared to control cells (WT) (**b**). (**F**) Mean ± SD relative viability of HCC cells cultured in serum free media (SFM) transfected with pEGFP-c2-*FOXO3a*-3A plasmid or Negative control; compared with negative control, ***P<0.001, two-way ANOVA.

To explore the potential role of FOXO3a in serum-deprived HCC cells, we artificially knocked down the expression of FOXO3a using a *FOXO3a*-specific siRNA. The efficiency of interference was confirmed by Western blotting (Figure 3A; WT vs. siRNA, **P < 0.01, *t*-test).

The cell count kit-8 assay demonstrated that cells transfected with negative control siRNA initially proliferated in the absence of 10% FBS; however, *FOXO3a*-siRNA interference reduced the growth curve and accelerated a decline in the viability of HCC cells in the absence of 10% FBS (Figure 3B (a); SFM + *FOXO3a*-siRNA vs. SFM + NC-siRNA, **P < 0.01 for Huh-7 and Hep3B, two-way ANOVA; SFM + *FOXO3a*-siRNA vs. SFM + NC-siRNA, **P<0.01 for HepG2, *t*-test). Interestingly, we did not observe the same effect for *FOXO3a*-siRNA in the presence of 10% FBS, as the growth curves of control cells and cells transfected with *FOXO3a*-siRNA were not significantly different in the presence of 10% FBS (Figure 3B (b); all *P*>0.05, two way ANOVA).

To further confirm the role of FOXO3a in HCC cells, we employed the overexpression pEGFP-c2-*FOXO3a*-3A plasmid encoding constitutively active from of FOXO3a (mutations in three AKT phosphorylation sites [32]) in our research. The efficiency of transfection was confirmed by Western blotting analysis (Figure 3E (b)). The CCK8 assay revealed that, compared to cells transfected with control vector, pEGFP-c2-*FOXO3a*-3A transfection significantly reversed the serum-deprivation-induced growth inhibition in HCC cells (Figure 3F; compared with Negative control, ***P<0.001, two-way ANOVA).

A EdU incorporation assay was used to estimate HCC cells proliferation. EdU is a nucleoside analog of thymidine, which incorporates into DNA during the synthesis of DNA [33]. We found that overexpressed active FOXO3a significantly attenuated inhibition of new DNA synthesis after 48h serum deprivation (Figure 3C, D; **P<0.01, *t*-test).Taken together, these results indicated FOXO3a plays a key role in maintaining proliferation of serum-deprived HCC cells.

### Serotonin upregulates FOXO3a in Huh7 cells, but not in HepG2 or Hep3B cells

**Figure 4:**
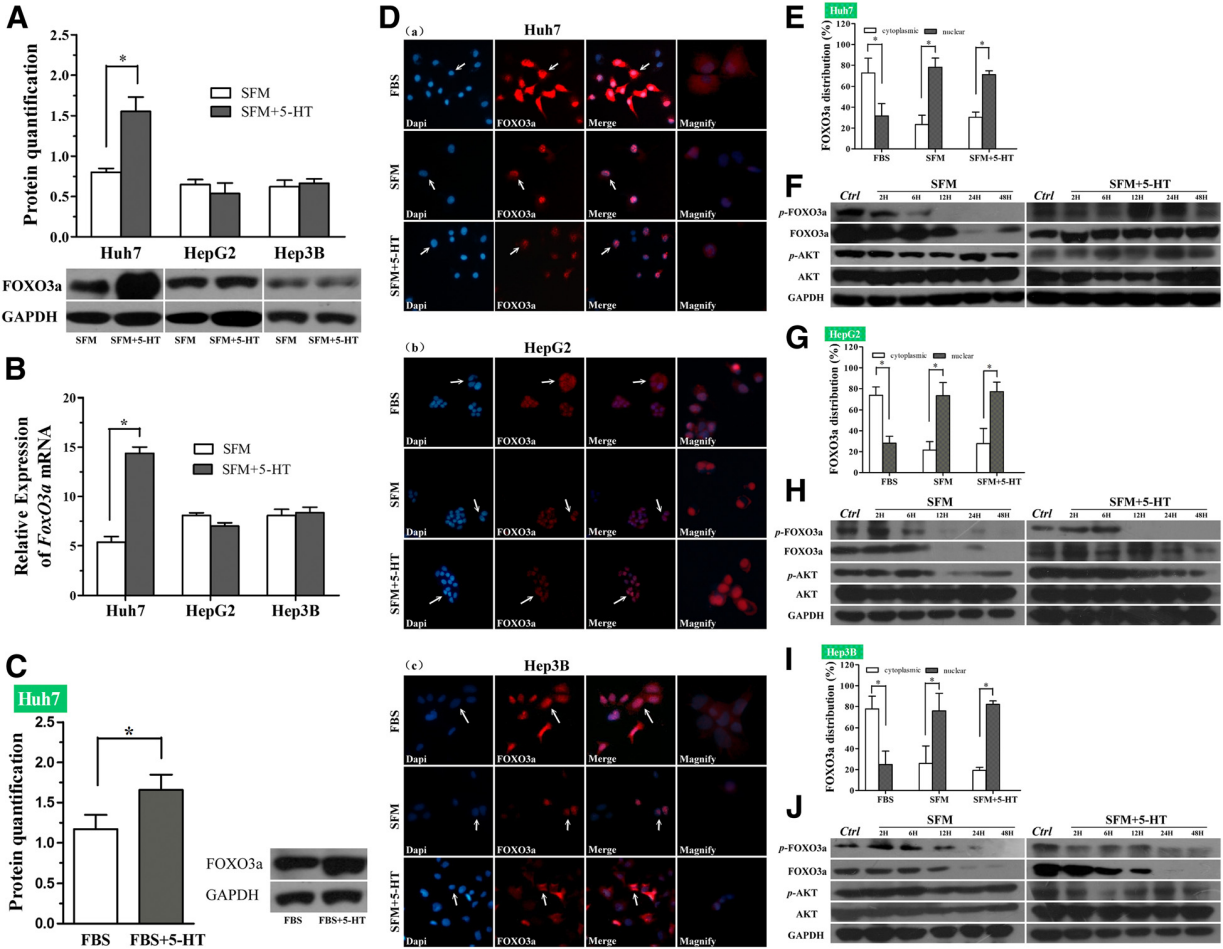
**Effect of serotonin on the expression and phosphorylation of FOXO3a in serum-deprived HCC cells.** (**A, B**) Western blot (**A**) and qRT-PCR analysis (**B**) of FOXO3a expression in HCC cells cultured in serum free media (SFM) with or without serotonin (5-HT) for 48h; *P<0.05, *t-*test. (**C**) Western blot analysis of FOXO3a expression in Huh7 cells cultured in media containing 10% fetal bovine serum (FBS) with or without 5-HT (50 μM) for 48h; *P<0.05, *t*-test. (**D**) The subcellular localization of FOXO3a in Huh7 cells (**a**), HepG2 cells (**b**) and Hep3B cells ( **c**) after 48h cultured in FBS, SFM or serum free media with serotonin (SFM+5-HT). Quantification of immunofluorescence staining patterns for FOXO3a in Huh7 cells (E), HepG2 (G) cells and Hep3B cells (**I**); *P<0.05, *t*-test. ( **F, H, J**) Western blot analysis of total-AKT, phospho-Akt (Ser473; *p*-AKT), total-FOXO3a and phospho-FOXO3a (Thr32; *p*-FOXO3a) in serum-deprived Huh7 cells (**F**), HepG2 cells (**H**) and Hep3B cells (**J**) cultured in the presence or absence of serotonin (5-HT) for the indicated times.

As serum deprivation led to reduced FOXO3a expression in HCC cells, we investigated whether FOXO3a was involved in the molecular mechanism by which 5-HT stimulates proliferation in serum-deprived Huh7 cells. As predicted, serotonin upregulated FOXO3a protein and mRNA expression in serum-deprived Huh7 cells (Figure 4A, B; SFM vs. SFM + 5-HT,*P < 0.05, *t*-test), but not in serum-deprived HepG2 or Hep3B cells. Additionally, serotonin also increased FOXO3a protein expression in Huh7 cells in the presence of FBS (Figure 4C; FBS vs. FBS + 5-HT, *P < 0.05, *t*-test;).

### Serotonin induces phosphorylation of AKT and FOXO3a in serum-deprived Huh7 cells, but not in HepG2 or Hep3B cells

To better identify the mechanism of regulation of FOXO3a mediated by serotonin in HCC cells, immunofluorescence staining was used to detect the subcellular localization of FOXO3a in HCC cells after cultured in media containing 10% FBS, serum free media with or without serotonin for 48h. We found that FOXO3a mainly located in cytoplasm in HCC cells in the presence of FBS, whereas FOXO3a predominately expressed in nuclei when HCC cells were cultured in serum free media with or without serotonin (Figure 4D, E, G, I). As phosphorylation of FOXO3a is a critical mechanism to regulate FOXO3a nuclear export, we also investigated the phosphorylation of FOXO3a and AKT in HCC cells cultured in serum free media in the presence or absence of serotonin. Western bolt analysis results revealed that serum deprivation caused decreased phosphorylation ratio of AKT and consequently reduced phosphorylation of FOXO3a in HCC cells in a time-dependently manner (Figure 4F, H, J; phosphorylation ratio of AKT and FOXO3a was not shown, see details in Additional file 1: Figure S1). Serotonin markedly reversed the dephosphorylation of AKT induced by serum deprivation and resulted in phosphorylation of FOXO3a in Huh7 cells, but not in HepG2 and Hep3B cells (Figure 4F, H, J; phosphorylation ratio of AKT and FOXO3a was not shown, see details in Additional file 1: Figure S1).

### FOXO3a mediates serotonin-induced proliferation in serum-deprived Huh7 cells

As serotonin significantly increased proliferation and upregulated FOXO3a in serum-deprived Huh7 cells, next we ascertained whether FOXO3a acted as a downstream target of serotonin. Huh7 cells were transfected with NC-siRNA or *FOXO3a*-siRNA, and cultured in SFM in the presence of 50 μM serotonin, and then cell proliferation was measured and the cell cycle was analyzed by flow cytometry.

**Figure 5:**
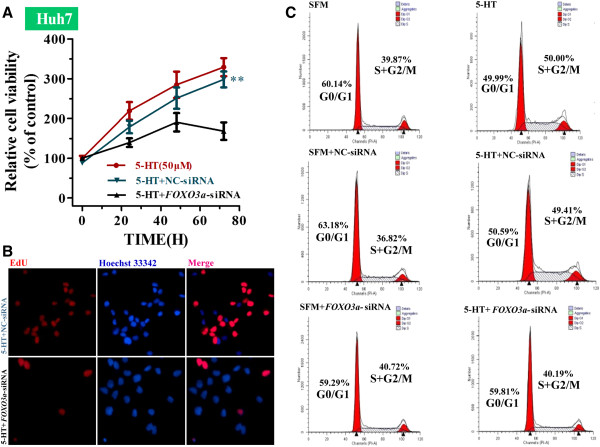
**Effect of serotonin and FOXO3a knockdown in serum-deprived Huh7 cells.** (**A**) Mean ± SD relative cell viability of serum-deprived Huh7 cells cultured in the presence of serotonin (5-HT) and transfected with Negative control siRNA (NC-siRNA) or *FOXO3a*-siRNA; 5-HT+NC-siRNA vs. 5-HT+ *FOXO3a*-siRNA, **P < 0.01, two-way ANOVA. (**B**) EdU staining patterns of serum-deprived Huh7 cells transfected with Negative control siRNA or *FOXO3a*-siRNA after 48h incubation with serotonin (5-HT).(**C**) Flow cytometry cell cycle analysis of serum-deprived Huh7 cells transfected with NC-siRNA, *FOXO3a*-siRNA or cultured with 5-HT.

*FOXO3a*-siRNA significantly reduced serum-deprived Huh7 cell proliferation in the presence of serotonin, compared with NC-siRNA-transfected cells (Figure 5A; 5-HT + *FOXO3a*-siRNA vs. 5-HT + NC-siRNA, **P < 0.01, two-way ANOVA). We also determined Huh7 cells proliferation by EdU incorporation assay. Consistent with CCK8 assay, *FOXO3a*-siRNA significantly inhibited DNA synthesis in Huh7 cells after 48h incubation in SFM in the presence of serotonin (Figure 5B).

As shown in Figure 5C, Huh7 cells incubated in SFM for 48 h exhibited a low rate of proliferation; however there was no significant difference in the S+G2/M phase cell cycle ratio of control cells (39.25 ± 1.36%), NC-siRNA transfected cells (38.62± 2.13%) and *FOXO3a*-siRNA transfected cells (39.73 ± 1.51%) in the absence of serotonin. In the presence of serotonin, the S+G2/M cycle ratio significantly increased in control cells (51.23 ± 1.25%) and NC-siRNA transfected cells (50.10 ± 1.72%); but not in *FOXO3a*-siRNA transfected cells (40.13 ± 0.96%). Serum-deprived cells transfected with *FOXO3a*-siRNA in the presence of serotonin displayed a similar rate of proliferation as control serum-deprived cells, confirming that knockdown of FOXO3a inhibited the ability of serotonin to induce proliferation in serum-deprived cells.

### Altered expression of the 5-HT2B receptor may regulate the response of serum-deprived HCC cells to serotonin

**Figure 6:**
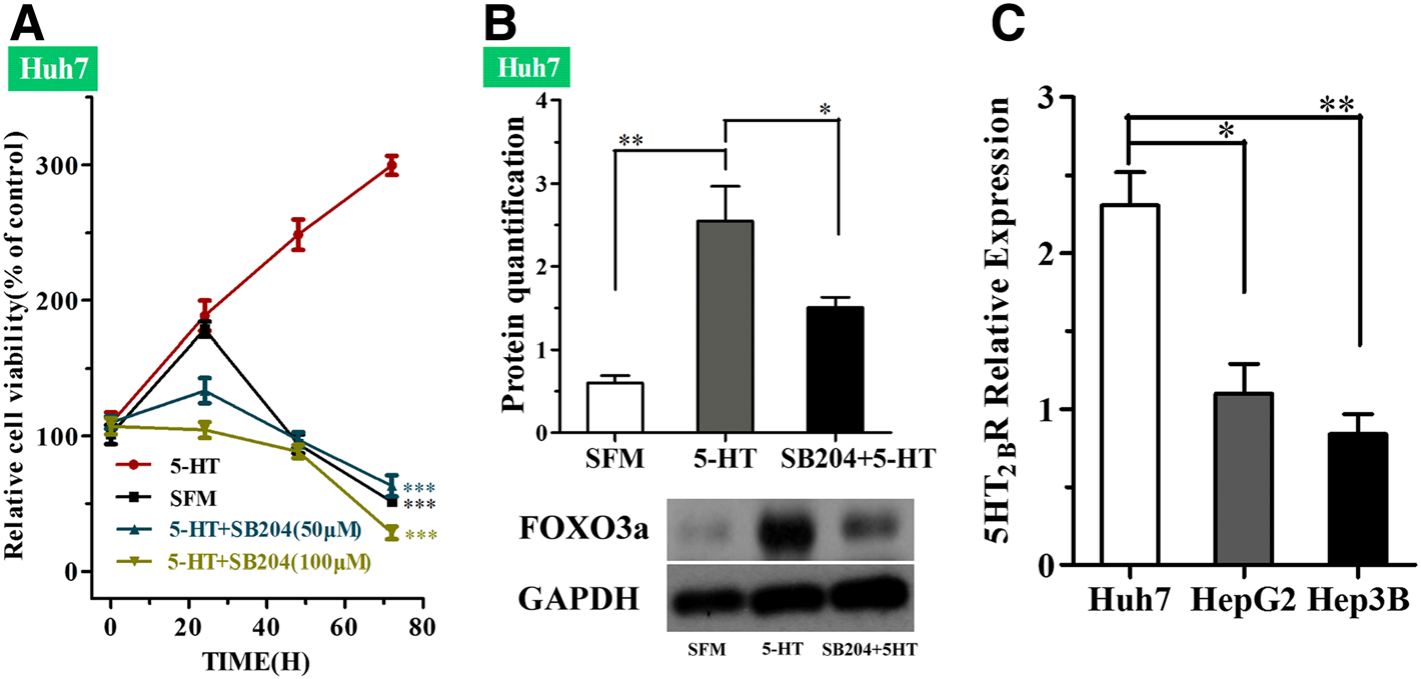
**Expression pattern of 5-HT**_**2B**_**R in HCC cells.** (**A**) Mean ± SD relative cell viability of serum-deprived Huh7 cells cultured in the presence or absence of serotonin (5-HT) and/or SB204; compared with 5-HT (50 μM), ***P < 0.001, two-way ANOVA. (**B**) Western blot analysis of FOXO3a protein expression in serum-deprived Huh7 cells after incubation with SB204 (50 μM) and serotonin (50 μM) for 48h; *P<0.05, **P<0.01, *t*-test. (**C**) qRT-PCR analysis of the basal expression levels of *5-HT*_*2B*_*R* mRNA in HCC cells; *P<0.05, **P<0.01.

Among the various types of serotonin receptor in human tissues, the 5-HT_2B_R is expressed at high level in hepatocytes. We investigated whether the regulation of FOXO3a by 5-HT was regulated via 5-HT_2B_R. Firstly, we determined the effect of the 5-HT_2B_R antagonist SB204 on the viability of Huh7 cells cultured in SFM in the presence of 5-HT. SB204 significantly reduced the cell viability of serum-deprived Huh7 cells, compared to cells cultured with 5-HT (50 μM) alone (Figure 6A; 5-HT (50 μM) vs. 5-HT+SB204 (50 μM), ***P < 0.001, two-way ANOVA), suggesting that serotonin regulates cell proliferation via 5-HT_2B_R.

As SB204 decreased the viability of serum-deprived cells, we examined whether SB204 affected FOXO3a protein expression. The addition of SB204 (50 μM) to serum-deprived Huh7 cells in the presence of 5-HT(50 μM) reduced the protein expression levels of FOXO3a (Figure 6B; 5-HT vs*.* SB204 + 5-HT, * *P* < 0.05, *t*-test).

In addition, we preliminarily investigated the mechanism by which the three HCC cell lines responded differently to serotonin. As the 5-HT2B receptor played a role in serum-deprived Huh7 cells, we investigated whether the mRNA expression levels of this receptor varied in different HCC cell lines. The basal expression levels of *5-HT*_*2B*_*R* mRNA were significantly higher in Huh7 cells than HepG2 and Hep3B cells (Figure 6C; Huh7 vs. HepG2, *P<0.05, *t*-test; Huh7 vs. Hep3B; **P < 0.01, *t*-test). This observation indicates that the higher expression levels of 5-HT_2B_R in serum-deprived Huh7 cells can more effectively mediate 5-HT-induced proliferation, compared to HepG2 or Hep3B cells where the 5-HT2B receptor is expressed at lower levels.

## Discussion

In addition to its function as a neurotransmitter in the CNS, serotonin, especially peripheral serotonin, was recently shown to promote the proliferation of many different cell types [2,5]. However, consensus on the mechanism by which serotonin acts as a mitogen had not yet been reached, and it was not known whether serotonin acts via a receptor or receptor-independent pathway, as the intracellular mechanisms underlying the mitogenic effects of serotonin are poorly characterized. In this study, we demonstrated that serotonin promoted the proliferation of serum-deprived HCC cells via the serotonin receptor 5-HT_2B_R and revealed that FOXO3a was a key downstream effector of serotonin.

The FOXO transcription factor family is evolutionarily conserved and plays important roles in development, differentiation, proliferation, apoptosis, stress resistance and metabolism [12,13]. Liang et al. reported that serotonin targets and regulates the activation of DAF-16, the *C. elegans* homologue of FOXO3a [34]. In mammals, serotonin can also regulate the expression of FOXO3a in the CNS [35]. However, the relationship between serotonin and FOXO3a in HCC cells is poorly characterized. Interestingly, we observed that FOXO3a was downregulated in serum-deprived HCC cells, and that knockdown of FOXO3a enhanced the sensitivity of HCC cells to serum deprivation and overexpression of active FOXO3a reversed the serum-deprivation-induced proliferative inhibition, indicating that FOXO3a is required for serum-deprived HCC cells to maintain normal growth. Furthermore, our data suggested that serotonin could reverse the serum-deprivation-induced inhibition of proliferation and G0/G1 phase cell cycle arrest, by increasing the expression of FOXO3a.

Function of FOXO3a is intimately associated with its post-translational modification (PTM), of which phosphorylation is the most important mechanism to regulate FOXO3a subcellular localization and its transcriptional activity [32]. In this study, we observed that serum deprivation inactivated AKT and led to retention of FOXO3a in nucleus. Serotonin had no effect on the subcellular localization of FOXO3a in serum-deprived HCC cells .However, serotonin increased the phosphorylation of AKT and FOXO3a in serum-deprived Huh7 cells, but not in HepG2 or Hep3B cells. Interestingly, the increased phosphorylation of FOXO3a induced by serotonin did not parallel cytoplasmic translocation of FOXO3a in Huh7 cells (Figure 4D (a)). The exact mechanism regulating nucleus/cytoplasm shuttling of FOXO3a is still unknown, phosphorylation modification is just one of many PTMs [36]. Moreover, a same stimuli may induce reverse modification of FOXO protein [37]. The phosphorylation of FOXO3a induced by AMP-activated protein kinase (AMPK) leads to accumulation in nuclear localization of FOXO3a [38]. In this study, we demonstrates that the promotion of serum-deprived HCC cells proliferation exerted by serotonin is mainly due to upregulation of FOXO3a in nucleus. However, further experiments are still required to clarify the precise mechanism of regulation of FOXO3a mediated by serotonin in HCC cells.

These results are seemingly in contrast with previous studies which reported that overexpression of FOXO3a induced cell apoptosis and cell cycle arrest, such as endothelial cells or follicular thyroid cancer cells, breast cancer cells and so on [14,39-41]. FOXO3a is a key intersection between various cell signaling pathways; however, the functions of FOXO3a and its target genes are poorly characterized. Increasing numbers of studies indicate that FOXO3a plays an important role in the initiation, progression and invasion of cancer [42,43]. Constitutively nuclear FOXO3a localization is believed to be associated with poor prognosis in breast cancer [44]. Storz et al. reported that FOXO3a promotes invasion in cancer due to serum deprivation [43]. FOXO3a also acts as a pro-survival factor in normal and cancer cells adapting to hypoxic stress, by inhibiting hypoxia inducible factor (HIF-1)-induced apoptosis [45]. Oh et al. reported that FOXO3a protects T-cells from apoptosis [46]. Lately, in agreement with our results, Li et al. reported that FOXO3a promotes tumor cell survival in serum deprivation [47].The underlying mechanisms which explain the varying responses of cells to FOXO3a have not yet been fully explored. The specific cell type, growth environment or target genes may all contribute to the different functions of FOXO3a. Although our results extend the knowledge of the function of FOXO3a, the existing knowledge does not yet form a complete picture of the diverse mechanisms of action of FOXO3a.

It is known that peripheral serotonin can mediate proliferation of cells in the hepato-GI tract [2,5,6]; however, the mechanism of this mitogenic effect was unknown. In this study, we proved that the serotonin receptor 5-HT_2B_R mediates serotonin-induced proliferation in serum-deprived Huh7 cells. Additionally, inhibition of 5-HT_2B_R in Huh7 cells using SB204, a unique antagonist of 5-HT_2B_R, significantly decreased the expression of FOXO3a, demonstrating that FOXO3a was the target of serotonin in Huh7 cells. Considering the irregular distribution and expression pattern of 5-HT_2B_R in human tissues, especially in tumor cells [3,4,9], it was of interest to note that different expression levels of 5-HT_2B_R could mediate a different response to serotonin in Huh7 cells (which express high levels of 5-HT_2B_R) compared to HepG2 and Hep3B cells (which express low levels of 5-HT_2B_R).

In addition to the effect of serotonin in serum free conditions, we also investigated the effect of serotonin in the presence of 10% FBS. Serotonin could increase the expression of FOXO3a in Huh7 cells cultured in 10% FBS, but could not enhance the rate of proliferation. Also, knockdown of FOXO3a in the presence of FBS had no significant effect on the rate of proliferation. These observations indicate that while FOXO3a is an indispensable downstream target of serotonin in serum-deprived cells, FOXO3a is not an essential component for cell growth in the presence of the other conventional growth-inducing polypeptides present in serum.

## Conclusion

In this study, we demonstrates that FOXO3a functions as a growth factor in serum-deprived HCC cells and serotonin promotes the proliferation of serum-deprived HCC cells via upregulation of FOXO3a, in the presence of sufficient levels of the serotonin receptor 5-HT_2B_R. Knockdown of FOXO3a enhanced the sensitivity of HCC cells to serum withdraw and inhibited cell growth. Targeting of the serotonin-5-HT_2B_R-FOXO3a pathway may be beneficial for liver cancer therapy.

## Competing interests

The authors declare that they have no competing interests.

## Supplementary Material

Additional File 1: Figure S1 The normalized ratios of phosphorylation of AKT and FOXO3a. The normalized ratios of phosphorylation of AKT and FOXO3a in Huh7 cells (A), HepG2 cells (B) and Hep3B (C) cells cultured in serum free media (SFM) with or without serotonin (5-HT) for the indicated times were calculated by analyzing the densities of Western bolt bands; compared with control (Ctrl), *P<0.05, *t*-test. Click here for file
