# Supplementary figures and images for: Serotonin promotes the proliferation of serum-deprived hepatocellular carcinoma cells via upregulation of FOXO3a
Source: Mol Cancer. 2013 Feb 19;12:14. doi: 10.1186/1476-4598-12-14 (PMC3601970; doi:10.1186/1476-4598-12-14)

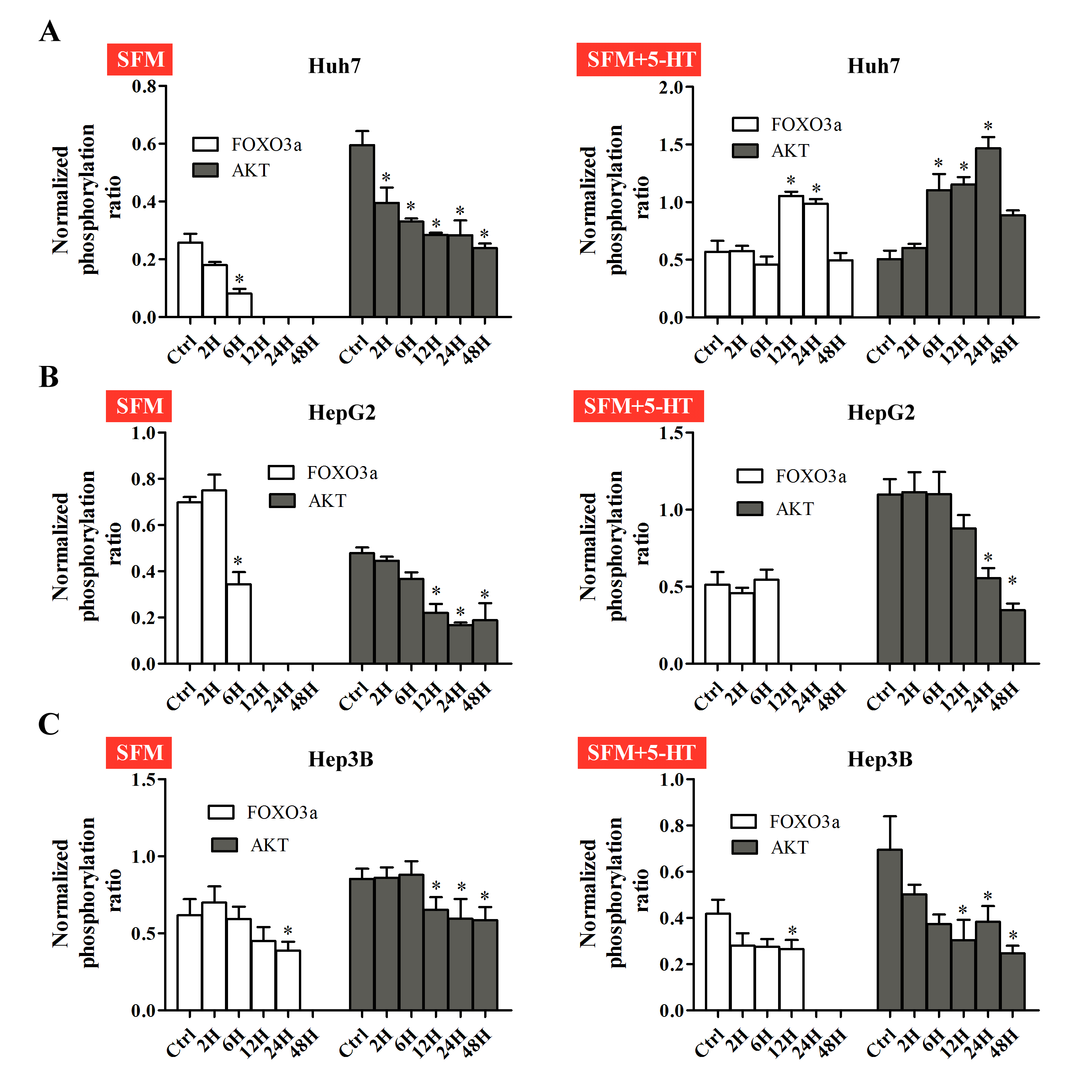

Supplement: Additional File 1: Figure S1 — The normalized ratios of phosphorylation of AKT and FOXO3a. The normalized ratios of phosphorylation of AKT and FOXO3a in Huh7 cells (A), HepG2 cells (B) and Hep3B (C) cells cultured in serum free media (SFM) with or without serotonin (5-HT) for the indicated times were calculated by analyzing the densities of Western bolt bands; compared with control (Ctrl), *P<0.05, t-test. [file 1476-4598-12-14-S1.tiff]
